# Measuring and Assessing Fluctuating and Authentic–Durable Happiness in Italian Samples

**DOI:** 10.3390/ijerph18041602

**Published:** 2021-02-08

**Authors:** Lucia Monacis, Pierpaolo Limone, Michaël Dambrun, Antonella Delle Fave, Maria Sinatra

**Affiliations:** 1 Department of Economics, Management and Territory, University of Foggia, 71121 Foggia, Italy; 2 Department of Humanities, University of Foggia, 71121 Foggia, Italy; pierpaolo.limone@unifg.it; 3 Université Clermont Auvergne, CNRS, LAPSCO, F-63000 Clermont-Ferrand, France; michael.dambrun@uca.fr; 4 Department of Pathophysiology and Transplantation, University of Milano, 20122 Milano, Italy; antonella.dellefave@unimi.it; 5 Department of Educational Sciences, Psychology, Communication, University of Bari, 70121 Bari, Italy; 6 University Institute SSML “N. Mandela”, 75100 Matera, Italy

**Keywords:** happiness, factor structure, measurement models, psychometric properties, convergent and discriminant validity, temporal stability

## Abstract

On the basis of the self-centeredness and selflessness model, a new instrument assessing two distinct dimensions of happiness, fluctuating and authentic–durable, was developed. The current research aimed at examining the factor structure of the Italian version of the instrument, its psychometric properties and construct validity. To this end, two studies were carried out. Study 1 (N = 544) examined different measurement models, from unidimensional to multidimensional, from a fully symmetrical bifactor solution to a bifactor (S-1) solution. Findings indicated better and adequate fit indices for the last model. Using various samples (n = 1274) Study 2 confirmed the bifactor (S-1) structure and analysed psychometric properties, convergent and divergent validity and temporal stability of the instrument. Findings generally gave evidence of the multidimensional conceptualization of the construct, good levels of reliability values and adequate convergent validity of both scales. Discriminant validity showed mixed results from no association of age with authentic–durable happiness to weak and negative association with fluctuating happiness. Test–retest reliability displayed an adequate value of correlation coefficient for the two set scores of the authentic dimension and a value below the recommended cut-off criteria for the fluctuating dimension, where significant differences in the mean scores emerged. Future studies should aim to replicate the results of this research and attempt to overcome its limitations.

## 1. Introduction

While traditional psychology paid more attention to the negative aspects of human behavior than to its positive aspects, the new expanding positive psychology movement has placed more emphasis on the study of the conditions and processes, which contribute to the optimal functioning of individuals, groups, and institutions [1]. Within this new understanding of psychology, well-being and happiness have emerged as main topics, although happiness has been treated as “an umbrella concept for notions such as well-being, subjective well-being, psychological well-being, hedonism, eudaimonia, health, flourishing, and so on” [2] (p. 3). Aside from the debate on a common and univocal definition of the concept, two relatively distinct views of happiness have been generally labelled: hedonism and eudaimonism. The former, which has its roots in Epicurean philosophy, reflects personal fulfilment of individualistic needs, the pursuit of positive emotion, seeking maximum pleasure with instant gratification [3,4]. The latter, whose origins date back to Aristotelian philosophy, was understood by Waterman [5] as the fulfilment or the realization of one’s daimon or true nature, and by Ryff and Keyes [6] as the realization of one’s true potential. According to Peterson, Park and Seligman, “uniting eudemonic emphases is the premise that people should develop what is best within themselves and then use these skills and talents in the service of greater goods” [7] (p. 26). In short, if the hedonic happy person “takes precedence over the happiness of the community”, the eudaimonic view promotes the integration of individual happiness with collective wellbeing [8] (p. 6).

Over the last few decades, a substantial amount of research has focused on psychological qualities that foster happiness measures according to different theoretical frameworks, from subjective well-being with the Subjective Happiness Scale (SHS-4) [9] including four items assessing the global assessment of one’s happiness, to psychological well-being with the Oxford Happiness Inventory (OHI-29) [10] comprising 29 items evaluating the general level and six domains of self-realization, or the Authentic Happiness Inventory (AHI-24 × 5) [11] consisting of 24 sets of five statements from which individuals have to choose the statement that best describes their feelings in the past week. The last instrument was based on the previous measures developed by Peterson and colleagues [12] that identified three routes/paths to happiness (life of meaning, life of pleasure and life of engagement). Dambrun and Ricard [13] elaborated the Self-centeredness/Selflessness Happiness Model (SSHM) taking into account the structure of the self and its related psychological functioning. Inspired by the interconnectedness of many disciplines, from western and eastern psychological to philosophical traditions, the model reviewed the traditional psychological dichotomies of independent/interdependent self and postulated self-centeredness and selflessness as two qualitatively distinct dimensions underlying psychological functioning. The first dimension is characterized by exaggerated self-interest, egoism, egocentrism, strong distinction between self and others, self and environment. Linked to hedonic principles, self-centered behaviors adopt an approach/avoidance mechanism, i.e., seeking pleasant and gratifying stimuli and avoiding unpleasant ones. Two effective reactions have emerged from this type of psychological functioning: transitory pleasure, in the sense of stimulus-driven pleasures, and afflictive effects, which are caused by the impossibility to obtain desired objectives and give rise to negative feelings, such as frustration, anger, hostility, jealousy and fear. The second dimension of psychological functioning is related to altruism, kindness, empathy, thus showing a strong feeling of connectedness between self and others. As selfless, prosocial-oriented behaviors share some core assumptions with eudaimonic tendencies, they are harmoniously interconnected with all the elements of a whole including oneself, the others and all living forms. The harmonious interconnectedness, strictly linked to benevolence affects not sensitive to environmental variations, enhances emotional stability and leads to self-transcendence. On these premises, this specific feeling is associated with the authentic–durable happiness characterized by two related dimensions, namely durable contentment and durable inner peace or plenitude. Conversely, the alternation of positive and negative phases, which is dependent upon circumstances and determined by the approach/avoidance mechanism, induces fluctuating happiness.

On the basis of the SSHM, Dambrun and colleagues [14] developed a new two-dimension instrument of the general construct of happiness: the Subjective Fluctuating Happiness Scale (SFHS) and the Subjective Authentic–Durable Happiness Scale (SA–DHS). The importance of this instrument may lie in the operationalization of the SFHS (perceived pleasure) and the SA–DHS (plenitude or inner-peace). The last dimension was already investigated in terms of durable contentment [15,16]. The dimensionality of the instrument was empirically tested by a series of confirmatory factor analyses [14]: what emerged were high internal consistency values, satisfactory test–retest validities, and adequate convergent and discriminant validities carried out with measures of well-being and mental health, as well as with other theoretically and empirically associated psychological constructs (presence of and search for meaning in life, optimism, mindfulness and sense of coherence).

On these premises, the first goal of the current research was to examine the factor structure of the Italian version of the instrument to provide further empirical evidence for its validity. For this purpose, different models (with one, two and three latent factors) were hypothesized. To shed more light on its factor structure, a bifactor model that captured one general factor and the other two factors were also assessed. In fact, in agreement with Tatarkiewicz’s statement that “happiness requires total satisfaction” [17] (p. 8), and in line with Dambrun and Ricard’s SSHM showing that the two above mentioned psychological mechanisms operate simultaneously along a single continuum, it was assumed that: (1) the factor durable contentment was the reference domain for the general (G) factor, given its unambiguous meaning related to general well-being and life satisfaction; (2) the fluctuating and the inner-peace dimensions were considered as the specific (S) factors within the bifactor model. 

The second goal sought to confirm the distinction between the SFHS and the SA–DHS by providing evidence of the convergent validity. Such examination was investigated using measures of well-being and happiness and some psychological constructs. In line with the eudaimonic perspective [18,19,20], well-being measures and psychological constructs were hypothesized to be positively associated with authentic–durable happiness and negatively associated with the fluctuating happiness. Moreover, since previous studies empirically demonstrated the positive association between the perceived presence of meaning in life and life satisfaction, optimism, and happiness [21,22], and the negative association of search for meaning in life with well-being measures [23,24], the SA–DHS was expected to be positively related to the presence of meaning in life and negatively related to search for meaning in life. On the contrary, the SFHS was supposed to be negatively related to the presence of meaning in life and positively to search for meaning in life. 

The discriminant validity of the instrument was also examined in relation to age. In line with [25] who affirmed there is no association of happiness with age, i.e., old and young are about equally happy in most countries, no significant or weak association of happiness constructs with age was expected. Finally, test–retest reliability was carried out to establish the consistency of the instrument across time, and consequently, to verify whether the scores were the same over two time-points. Thus, high relationships between the two data set points of each dimension of the scale were expected.

## 2. Materials and Methods–Study 1

### 2.1. Participants and Procedure

A random sample of 565 high school and undergraduate university students meeting some practical criteria, such as geographical proximity and easy availability at a given time, was recruited using convenience sampling. Twenty-one cases with more than 10% missing data were deleted during cleaning the dataset. The final sample was composed by 544 participants (M*_age_* = 24.16 years, SD = 12.89; 283 females and 261 = males; 258 = adolescents and 286 = adults). The study was conducted in accordance with the ethical principles described by the Declaration of Helsinki. Written informed consent was obtained from the respondents and parents for adolescents up to 18 years. Respondents were invited to take part in the study by completing anonymously and voluntarily a self-report questionnaire that took on average 20 min. Data were collected from September to November 2019.

### 2.2. Measure

The first section included demographic questions concerning gender and age. The Italian version of the instrument composed by the Subjective Fluctuating Happiness Scale (SFHS) and the Subjective Authentic–Durable Happiness Scale (SA–DHS) [14] was used (Appendix A). The first scale comprising 10 items rated on a 7-point Likert scale (from 1 = Strongly disagree to 7 = Strongly agree), is designed to assess how much participants agree or disagree with each item. Examples of items are: “I have had satisfactions and also great disappointments” (item 1) and “In the same day, I can sometimes be happy and sometimes sad” (item 10). A single composite score was computed by averaging responses to all items. Higher scores reflect greater subjective fluctuating happiness. The second scale includes 16 items: 13 items assess authentic–durable happiness and three assess durable unhappiness. The last three items control for the compliance bias. Participants indicate their permanent level of happiness using a 7-point Likert scale from 1 = Very low to 7 = Very high. Examples of items are: “Satisfaction” (item 6) and “Fulfilment” (item 11). A single score was calculated by averaging 13 items. Higher scores reflect greater authentic–durable happiness. The scale consists of two sub-scores, the contentment component with eight items evaluating overall well-being, happiness, pleasure, bliss, satisfaction, beatitude, fulfilment and joy, and the inner-peace dimension with five items assessing peace of mind, serenity, inner-peace, inner-calm/tranquillity and plenitude. The instrument was translated from English into Italian separately by the Italian authors of this study. The resulting Italian version was then back-translated into English by a native speaker to establish comparability and to resolve any discrepancies.

### 2.3. Data Analysis

Before performing the data analysis, cleaning of the dataset was conducted by the inspection of the univariate normality and the multivariate outlines. As a result of these procedures, no case was removed. First, descriptive statistics analyses implying independent-samples t-tests were computed to verify gender and age effects on the scores of both scales. Second, data were submitted to confirmatory factor analysis (CFA) to assess the factor structure of the instrument, i.e., unidimensional, two factors, three factors and bifactor solution. CFA was performed with the Robust maximum likelihood (MLR) estimation since it is robust to violations of the assumption of normality [26,27]. The χ^2^ and its degrees of freedom (df), the comparative fit index (CFI), the root mean square error of approximation (RMSEA) plus its 90% confidence interval (CI), and the standardized root mean square residuals (SRMR) were used. For χ^2^, test values associated with *p* > 0.05 were considered good-fitting models; for CFI, values greater than or equal to 0.90 were accepted as indicators of good fit [28]. Hu and Bentler [29] demonstrated that RMSEA is one of the most informative criteria and recommended a value close to 0.06 in conjunction with an SRMR value of 0.08 or less. A *p*-value of 0.001 was set as the critical level for statistical significance. All statistical analyses were performed using Mplus 7.2 and SPSS for Windows 20.0.

### 2.4. Results

Descriptive statistics (means and standard deviations) of the variables of interest for the total sample and for gender groups are shown in Table 1. Significant age difference emerged in the sub-score of the contentment component, t(542) = 2.465, *p* < 0.050. More specifically, adolescents obtained higher scores. As for gender significant effects were found in fluctuating happiness score, t(542) = −4.887, *p* < 0.000, authentic–durable happiness, t(542) = 3.883, *p* < 0.000, and in the two sub-scores, i.e., contentment dimension, t(542) = 3.636, *p* < 0.000, and inner-peace dimension, t(542) = 3.668, *p* < 0.000. In other words, females obtained higher scores in the fluctuating dimension, whereas males in the authentic–durable happiness and in the two sub-scores. In addition, age differences within gender groups were found: adolescent males obtained higher scores in the dimensions of authentic–durable happiness, t(259) = 2.599, *p* < 0.000, and the two sub-scores, i.e., contentment dimension, t(259) = 2.709, *p* < 0.000, and inner-peace dimension, t(259) = 2.092, *p* < 0.050, whereas adult females obtained higher scores in the inner-peace dimension, t(281) = −2.244, *p* < 0.050.

In the first step, three prominent CFA models were performed. They are shown in Table 2. Model 1 evaluated the single-factor structure. The results indicated that the scale did not fit the model well. Model 2 provided the CFA analysis of the model with the two-factor construct. The first latent factor reflected the items of the fluctuating happiness scale (SFHS) and the second the items of the authentic–durable happiness scale (SA-DHS). Poor fit indices emerged. Model 3 was tested to evaluate the three-factor solution with the first latent factor reflecting the same items of the SFHS, the second factor comprising the items of the first component of the SA-DHS, i.e., the contentment dimension, and the third latent factor including the second component of the SA-DHS, i.e., the inner-peace dimension. The last two dimensions were correlated, since they captured the same latent variable of the SA-DHS. Findings showed inadequate fit indices, although both RMSEA and CFI values indicated improvement in fit. In a second step, the first-order CFA models were specified as a fully symmetrical bifactor model (Model 4) and as a bifactor-(S-1) version (Model 5). Model 4 assumed a General factor (G) as a general trait of the construct, and the three Specific factors (S) as specialities of certain trait domains, i.e., fluctuating, contentment, and inner-peace. Although results yielded adequate fit indices, a careful inspection indicated some anomalous data. This is in line with what Eid and colleagues previously affirmed in relation to nonsignificant S or G loadings, idiosyncratic negative loadings, or nonsignificant variances of S factors [30]. Indeed, results from Model 4 indicated that some observed parameters were not in line with the idea of a fully symmetrical bifactor model. As for the specific domain of contentment, two nonsignificant factor loadings (items 6 and 11) and one very small factor loading (item 4 < 0.200 level of significance) were found. Regarding the second specific domain of fluctuating, two very small factor loadings (related to items 9 and 4 < 0.200 level of significance) were observed. Finally, the G factor included a nonsignificant loading (item 1). Consequently, the fully symmetrical modeling-approach was suboptimal and the domains emerged as structurally different. To overcome this limitation, the bifactor (S-1) approach [30] was used: the contentment dimension was considered as the reference domain for the G factor (General Reference Factor), the inner-peace and the fluctuating dimensions were considered as S factors (Specific Residual Factors). All specific factors were defined to be orthogonal. Findings of Model 5 yielded adequate fit indices, thus this model was retained for further examination. The standardized GRF factor loadings of the items were significant and ranged from λ = −0.106 to λ = 0.834. The standardized SRF factor loadings ranged from λ = 0.341 to λ = 0.727 (*fluctuating*) and from λ = 0.392 to λ = 0.544 (*inner-peace*). The negative association between the two S factors was significant (*r* = −0.263).

## 3. Participants and Procedure–Study 2

The assessment of the bifactor model (S-1), reliability and validity of the instrument was examined using four samples. Sample 1 comprising 394 university students (M*_age_* = 25.01 years, SD = 8.53; 208 females and 186 males) was used to replicate and confirm the bifactor structure and reliability. Sample 2 including 254 young adults (M*_age_* = 24.87 years, SD = 7.90; 139 females and 115 males) was used to examine convergent validity by taking into account some psychological constructs, namely optimism, presence of and search for meaning in life, and mindfulness, which have been theoretically and empirically associated to happiness, as already reported in above-mentioned studies. Sample 3, composed of 488 participants (M*_age_* = 18.39 years, SD = 5.425; 268 females and 220 males; 367 adolescents and 121 adults), was used to analyse convergent validity between this instrument and other related measures of happiness and well-being. Sample 4, which recruited 138 young adults (M*_age_* = 32.75 years, SD = 12.57; 77 females and 61 males), was used to evaluate the temporal stability of the scales over a 12 weeks period. 

Data were collected at different times, from December 2019 to July 2020. In the first phase, participants were invited to complete anonymously and voluntarily a paper–pencil self-report questionnaire that took on average 20 min. Verbal informed consent form was obtained from all adult participants and from parents for students aged below 18 years. Individuals aged over 18 years were recruited from different university courses. Participants fulfilled the questionnaire during regular traditional lessons. During the pandemic period the online data collection methodology via snowball sampling technique was obviously adopted because of the social limitations and restrictions imposed by the Italian government. An online version of the questionnaire was provided to adolescents and young adults through a survey link. The study was conducted in accordance with the ethical principles described by the Declaration of Helsinki.

### 3.1. Measures

The questionnaires included demographic questions concerning gender and age, the two scales of happiness and a battery of self-reported scales. The Italian version of the Subjective Fluctuating Happiness Scale (SFHS) and of the Subjective Authentic–Durable Happiness Scale (SA-DHS) [14] was used. Cronbach’s values were good for both scales: 0.857 for the SFHS, 0.926 for SA-DHS. The psychological constructs were assessed through the following measures: (1) dispositional optimism assessed by the Life Orientation Test (LOT-R) [31] composed of ten items rating on a 5-point Likert scale (from 1 = strongly disagree to 5 = strongly agree). Three items are positively worded, whereas three items are negatively worded. The remaining four items are filler. High scores of six items indicate expectancies for future positive events. Internal consistency showed good value (0.735); (2) presence of and search for meaning in life measured by the Meaning of Life Questionnaire (MLQ) [32], which is composed of ten items measured through two 5-item subscales: Presence of Meaning (POM), i.e., the cognitive component referring to the degree to which individuals comprehend and perceive their lives as significant and purposeful, and the Search for Meaning (SFM), i.e., the motivational component indicating the level of individuals’ active engagement in establishing their comprehension of the meaning and purpose of their lives. Each scale is rated on a 7-point Likert scale from 1 (*absolutely untrue*) to 7 (*absolutely true*). Item 9 belonging to the POM subscale is reverse coded. Two total scores are calculated with high value indicating higher levels of the constructs. Internal consistency showed a good value equal to 0.866 for SFM and 0.821 for POM; (3) mindfulness assessed by the Mindful Attention Awareness Scale (MAAS) [33] comprising 15 items rated on a 7-point Likert scale from 1 (*almost always*) to 7 (*almost never*). The instrument assesses individual differences in the frequency of mindful states over time. Respondents indicate how frequently they happen to feel any of the experiences described. A total score is computed with a higher value indicating a higher level of mindfulness. Internal consistency showed a good value (0.849).

The measures of happiness and well-being were assessed by (i) the Satisfaction with Life Scale (SWLS) [15], which is designed to assess global cognitive judgments of individuals’ life satisfaction. Respondents are asked to indicate how much they agree or disagree with each of the five items using a 7-point Likert scale (from 1 = *strongly disagree* to 7 = *strongly agree*). Examples of items are: “In most ways my life is close to my ideal”, “I am satisfied with my life”. Cronbach’s α was good (=0.836); (ii) the Positive Affectivity and Negative Affectivity Schedule (PANAS) [34]. The instrument comprises two 10-item mood scales that measure two uncorrelated factors, namely ‘Positive Affect’ and ‘Negative Affect’, in terms of dispositional or trait affect, emotional fluctuations throughout a specific period of time, or emotional responses to events. Ten items measure Positive Affect (e.g., *enthusiastic*) and 10 Negative Affect (e.g., *scared*). Respondents are asked to rate the extent to which they experienced each mood, using a 5-point Likert scale (from 1 = *not at all* to 5 = *extremely*). The Cronbach’s α values were 0.854 for the Positive Affect Scale and 0.812 for the Negative Affect Scale, revealing very good internal consistency; (iii) the Oxford Happiness Inventory (OHI) [35], which measures positive psychological functioning. The instrument is made up of 29 items ranged on a 4-point response scale. Respondents are invited to choose one of four alternatives sentences concerning mastery and self-fulfilment, satisfaction with life, vigour, social interest and social cheerfulness. The sentences reflect incremental statements from 1 (*I do not feel happy*) to 4 (*I am incredibly happy*). An example of an item is: “I am not particularly optimistic about the future/I feel optimistic about the future”. The total scale had an overall high internal reliability of 0.914; (iv) the Flourishing Scale (FS) [36] and the (v) Scale of Positive and Negative Experience (SPANE) [36]. The FS is an eight-item scale concerning the cognitive component of subjective wellbeing. Indeed, it relates to the eudaimonic aspects of well-being, namely individuals’ optimal psychological functioning (e.g., meaning in life, positive relationships, and self-acceptance). Items are rated on a 7-point Likert scale from 1 (*strongly disagree*) to 7 (*strongly agree*). Examples of items are: “I lead a purposeful and meaningful life”, “I am optimistic about my future”. The total score is calculated by the sum of the item scores, with higher scores meaning that the respondent rates himself/herself as a very positive functioning individual. Cronbach’s α for the scale was 0.885, indicating high internal consistency. The SPANE is a 12-item scale made up of two subscales: six items for positive affect and six items for negative affect. It focuses on hedonic wellbeing, a construct that corresponds to the presence of life satisfaction and positive affect, and the absence of negative affect. The scale uses adjectives with a general significance, with positive valence such as “good”, “positive”, “pleasant”, and negative valence such as “bad”, “negative”, “unpleasant”. Respondents rate how often they had experienced in the previous month the feelings indicated by each item rated on a Likert-type scale ranging from 1 (*very rarely or never*) to 5 (*very often or always*). Two total scores are calculated for each effect. The Cronbach *α* of the scale was good, equal to 0.816 for the Positive Affectivity score and to 0.803 for the Negative Affectivity score. 

### 3.2. Data Analysis

CFA was performed to replicate and confirm the bifactor model (S-1) of the instrument. The same indices and criteria for the model fit were used. In addition, following Rodriquez and colleagues’ methodological suggestions [37] further statistical indices were analysed to examine the reliability and validity of the construct of happiness. The reliability was assessed by (a) omega reliability coefficients, ω and ωS, which reflect the systematic variance affecting unit-weighted composite scores attributable to multiple common factors. Higher values indicate a highly reliable multidimensional composite; (b) as indices of factor reliability, hierarchical omega coefficients, ωH and ωHS, which reflect the proportion of variance in scores attributable to the general reference factor as well as the proportion of variance in the scores explained by each specific residual factor after removing variance explained by the general reference factor [38]. The high value of omega H (>0.800) indicates that the total scores can be considered essentially unidimensional in the sense that the vast majority of reliable variance is attributable to a single common source [39]; (c) the relative omega for the GRF, which refers to the percent of a reliable variance (PRV) in the multidimensional composite due to the general reference factor, and for the SRF, which corresponds to the proportion of reliable variance in each subscale composite that is independent of the GFR. Other ancillary bifactor measures related to the factor level were considered, i.e., Explained Common Variance (ECV S&E) and Explained Common Variance (ECV New). They indicate the degree to which parameter estimates are biased when multidimensional constructs are forced into a unidimensional model. In addition, at the item level, the Item-level ECV (I-ECV) was also considered, because it serves as an assessment of unidimensionality and indicates the extent to which the responses to an item are accounted for by variation on the latent general dimension. According to Stucky and Edelen [40], selection items with large loading on the general factor and I-ECV values greater than 0.80 or 0.85 typically yield a fairly unidimensional set that reflects the content of the general dimension (p. 202). At the model level, Percentage of Uncontaminated Correlations (PUC) was interpreted along with ECV and served as an indicator of the percentage of Contentment item correlations contaminated by variance attributed to the general and domain-specific factors. According to Rodriguez et al. [37], ECV and PUC reflect the degree to which parameter estimates are biased when multidimensional constructs are forced into a unidimensional model. When ECV and PUC are greater than 0.700, the common variance within a model can be regarded as essentially unidimensional. As for the validity of the construct, the construct replicability index (H), i.e., the degree to which a factor is well defined by its indicators, and the factor determinacy index (FD) implying the correlation between factor scores and the factors were also calculated. The convergent validity for the two measures of happiness and for each component of the SA-DHS was analysed using their associations with other well-being and happiness measures and various psychological constructs. The discriminant validity was examined using patterns of association with age. Test–retest reliability was assessed to determine the consistency of a test across time, i.e., to verify whether the scores were the same over two time-points. 

### 3.3. Results

#### 3.3.1. Model Estimation

**Figure 1:**
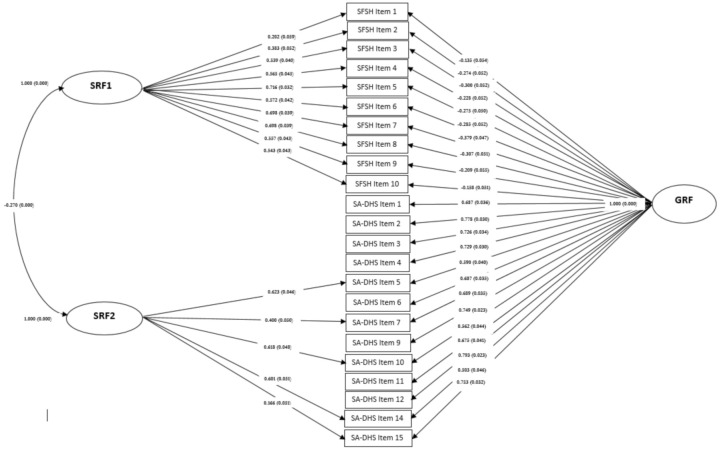
Path diagram of the bifactor solution GRF = General Reference Factor, Contentment; SRF1 = Specific Residual Factor, Fluctuating factor; SRF2 = Specific Residual Factor, Inner-peace.

Fit indices of the bifactor model were acceptable, χ^2^ = 612.645, df = 214, *p* < 0.001, CFI = 0.895, RMSEA = 0.069; 90% C.I. 0.062–0.075, SRMR = 0.049, and all factor loadings loaded significantly onto the GRF (*p* < 0.050). Table 3 reports the standardized loadings of the GRF, ranging from λ = 0.657 to λ = 0.793 for contentment factor, and the SRF factor loadings ranged from λ = 0.202 to λ = 0.716 for *fluctuating* factor and from λ = 0.166 to λ = 0.623 for *inner-peace* factor. All factor loadings were significant (all *ps* < 0.001, except for the last item of the second factor *ps* < 0.050). Specific factor loadings showed a fairly homogenous pattern across dimension for each source. The moderate and negative association between the two SRF was significant (*r* = −0.270). Figure 1 shows the bifactor model.

#### 3.3.2. Bifactor Model-Based Reliability

The results shown in Table 4 indicated that the omega total (ω) was 0.880. Thus, the general reference factor and the specific latent subscales could explain 88% of the total variance of all items in the extended bifactor model (S-1). In other words, only 12% of the variance was estimated due to random error (uniqueness) not explained by the model. Omega hierarchical (ωH) was 0.520, thus, 52% of the variance in total scores could be attributed to the individual differences on the general reference factor as a single general latent factor. This value suggested that the general reference factor could be considered as a broad general latent trait, since it was above 0.050 as recommended by Reise and colleagues [41]. About 36% of the variance in total scores was estimated to be due to specific factors. Thus, raw total scores of the general reference trait have to be interpreted as appropriate and as a distinct dimensional reflection of the general trait construct of happiness. 

When looking at the subscale level, values of the hierarchical omega coefficients (HωS) were reduced due to the large effect of the general reference factor and they were lower compared to the corresponding omega values (ωS). Indeed, the omega hierarchical subscale and omega subscale for SFHS were 0.308 and 0.857, respectively. This meant that the SFHS items tended to load higher on their corresponding subscale than on the general reference factor. In other terms, this indicator exhibited strong specificity for the interested factor. The omega hierarchical subscale and omega subscale for inner-peace were 0.055 and 0.895, respectively. Likewise, the inner-peace items tended to load higher on their corresponding subscale than on the general reference factor. This indicator showed strong specificity for its factors. Results from the relative omega coefficients for the general reference factor score and for subscales scores representing the percentage of reliable variance (PRV) in the multidimensional composite showed a good level in using the scores of the general reference factor and of the first specific residual factor, i.e., fluctuating happiness, and an acceptable level for the second specific residual factor score, i.e., the inner-peace, as suggested [42]. This evidence supported the multidimensionality of the instruments.

At the item level, all items of the fluctuating factor demonstrated a domain-specific content, as evidenced by I-ECV values ≤ 0.340, whereas some items of the inner-piece factor collapsed in the general reference factor. All items of the general reference factor exhibited I-ECV values equal to 1.00, thus reflecting the unidimensionality. At the model level, being values of ECV for the general reference factor and of PUC equal to 0.610 and 0.700, respectively, thus below the above-mentioned cut-off criteria, the multidimensional rather than unidimensional conceptualization of the happiness measurement was supported. Regarding the construct validity, H indexes showed high values for the general reference factor and for one of the specific factors (>0.700) [43], thus indicating that the two latent variables may be considered well defined by items. The second specific factor, inner-peace, gave evidence of almost adequate construct replicability. Moreover, according to Gorsuch’s [44] recommendations, factor score estimates may be used only when FD is greater than 0.900: for the GRF the estimate was 0.960 and for the two SRF values ranged from 0.930 to 0.870.

#### 3.3.3. Convergent Validity

The convergent validity for the two measures of happiness and for each component of the SA-DHS was analysed by using zero-order and partial correlations.

#### 3.3.4. SA-DHS and SFHS and measures of well-being and happiness

In sample 2 and sample 3, findings from bivariate correlations showed that the SA-DHS was associated positively with the emotional positive components of subjective well-being, positive experience and positive affectivity (*r* ranged from 0.112 to 0.703), and negatively with the negative components of subjective well-being, negative experience and negative affectivity (*r* ranged from −0.140 to −0.478). In the case of the SFHS, findings indicated that this dimension was associated negatively with the positive components of subjective well-being (*r* ranged from −0.176 to −0.472), and positively with the negative components of subjective well-being (*r* ranged from 0.291 to 0.463). Both scales correlated significantly with life satisfaction; negatively for the SFHS (*r* ranged from −0.183 to −0.362), and positively for the SA-DHS (*r* ranged from 0.149 to 0.705). Likewise, the total score of OHQ correlated with both scales, negatively for the SFHS (*r* ranged from −0.157 to −0.317), and positively for the SA-DHS (*r* ranged from 0.587 to 0.640). Finally, the measure of eudaimonic well-being, i.e., flourishing, correlated positively with the SA-DHS (*r* ranged from 0.128 to 0.611) and negatively with the SFHS (*r* ranged from −0.133 to −0.269). Partial correlations were also performed. First, the associations between SA-DHS and the other well-being measures were controlled for the SFHS, and second, the associations between SFHS and the other well-being measures were controlled for the SA-DHS. While the positive relationships of the SA-DHS with the positive components of subjective well-being were confirmed to be significant, its negative relationships with the negative components of subjective well-being were only partially confirmed to be significant. Similarly, the positive associations of the SFHS with the negative components of subjective well-being were confirmed to be significant, whereas its negative associations with the positive components of subjective well-being were partially supported. Findings indicated a significant trend of the negative association of life satisfaction with SFHS, but the trend of its positive association with SA-DHS was not confirmed. The total score of OHQ and both dimensions of happiness were found to be appropriately correlated. This appropriate correlation was also found in the associations of flourishing with both scales. (Table 5).

#### 3.3.5. SA-DHS and SFHS and measures of psychological constructs

Bivariate and partial correlations were also carried out to assess the convergent validity between the two scales of happiness and optimism, presence of and search for meaning in life, and mindfulness. Table 6 shows the main findings in the total sample, in males and females. As expected, in the case of the bivariate correlations, moderate coefficients emerged in the negative SFHS-optimism association (*r* ranged from −0.347 to −0.486) and in the positive SA-DHS-optimism association (*r* ranged from 0.349 to 0.434); from weak to moderate coefficients were found in the negative SFHS-POM association (*r* ranged from -0.253 to −0.310) and in the positive SA-DHS-POM association (*r* ranged from 0.411 to 0.476); weak coefficients resulted in the positive SFHS-SFM association (*r* ranged from 0.136 to 0.292) and in the negative SA-DHS-SFM association (*r* ranged from −0.114 to −0.199), and similarly, in the negative SFHS-MAAS association (r ranged from −0.231 to −0.342) and in the positive SA-DHS-MAAS association (*r* ranged from 0.189 to 0.288). In the case of the partial correlations, findings generally corroborated previous analyses. Individuals who were optimist, mindful and perceived their lives as significant and purposeful, experienced a more authentic–durable happiness and less fluctuating happiness, whereas those who searched for meaning in their life, experienced only fluctuating happiness. A careful inspection of the partial coefficients revealed that the negative association between the search for the meaning of life and authentic–durable happiness became non-significant. Consequently, this psychological construct has not to be considered as a predictor of authentic–durable happiness, it is only linked to increasing fluctuating happiness. (Table 6)

#### 3.3.6. Distinguishing the two components of the SA-DHS

The zero-order correlations between each score of the SA-DHS with measures of happiness, well-being and psychological constructs were run (Table 5, Table 6 and Table 7). In general, findings confirmed that contentment and inner-peace were related positively with happiness and wellbeing measures and negatively to the negative dimension of eudaimonic well-being. Participants generally reported higher mean scores on contentment subscale (M = 4.555, SD = 1.187) than on inner-peace subscale (M = 4.402, SD = 1.199; t(1273) = 6.486, *p* < 0.001). Table 5; Table 7 also report the partial correlation coefficients. When the inner-peace dimension was controlled for, the contentment dimension was highly related negatively to the negative components of subjective wellbeing (negative emotions and negative affectivity) and positively to well-being measures (positive affectivity, flourishing and life satisfaction) and to the psychological construct of dispositional optimism. No significant associations of contentment with the two components of meaning of life and mindfulness were found. By contrast, when contentment was controlled for, results confirmed the significant and positive connection of the inner-peace dimension with positive emotions and the presence of meaning in life. When comparing the partial coefficients that emerged in the associations between the two dimensions of SA-DHS with happiness and mindfulness, data showed similar positive and significant results in the case of happiness and the absence of significant relations in the case of mindfulness. These findings displayed that the contentment dimension was more closely related to positive affectivity, negative emotions, negative affectivity and flourishing in the total sample and to optimism in males, whereas the inner-peace dimension appeared to be more closely to positive emotions and the presence of meaning of life. Finally, both subscales seemed to be similarly linked to happiness and life satisfaction.

#### 3.3.7. Discriminant Validity

Bivariate correlations were performed across the four samples between the two measures of happiness and age as a discriminant variable. Findings emerged across the samples partially confirmed the expected hypotheses. Indeed, a negative association of age with the SFHS (*r* = −0.135, *p* < 0.000) and no association with the SA-DHS (*r* = 0.043, *p* > 0.05) were resulted. A careful inspection of findings showed that in the sample of the first study and in samples 3 and 4 in time 2, age was neither associated with the SFHS (*r* = −0.014, *p* > 0.100; *r* = −0.089, *p* > 0.050; *r* = −0.119, *p* > 0.100) nor with the SA-DHS (*r* = 0.028, *p* > 0.100; *r* = 0.004, *p* > 0.100; *r* = 0.015, *p* > 0.100), respectively. Conversely, in sample 1, sample 2 and sample 4 in time 1 of the second study, mixed results were found ranging from a weak association of age with the SFHS (*r* = −0.116, *p* < 0.050; *r* = −0.205, *p* < 0.050; *r* = −0.215, *p* < 0.050) to no significant association with the SA-DHS (*r* = 0.057, *p* > 0.100; *r* = 0.014, *p* > 0.100; *r* = 0.005, *p* > 0.100).

#### 3.3.8. Test-Retest Reliability and Agreement

The temporal stability of the instrument was carried out in sample 4 over a 12 weeks period. Correlation coefficients of the two set scores of the SA-DHS (*r* = 0.710, *p* < 0.001) and of the SFHS (*r* = 0.625, *p* < 0.001) indicated an adequate reliability, although the second value was below the recommended value of 0.700. However, when looking at the mean score of the two scales between the two intervals, scores on SFHS were significantly different between time 1 (M = 4.426, SD = 1.270) and time 2 (M = 4.038, SD = 1.209), t(137) = 3.391 *p* < 0.010), whereas scores on SA-DHS were not significantly different in time 1 (M = 4.481, SD = 1.298) and time 2 (M = 4.641, SD = 1.286), t(137) = −1.334 *p* > 0.100).

## 4. Discussion

The aim of the current research was twofold: (i) to analyze the factorial validity by examining the measurement model and the psychometric properties of the Italian version of the instrument assessing two distinct dimensions of happiness; (ii) to provide empirical evidence of the construct validity by evaluating convergent and discriminant validity and temporal stability. To this end, two studies were carried out. Study 1 examined the multidimensional factor structure of the Italian version. Confirmatory factor analyses were carried out to scrutinize different measurement models: from unidimensional with one single latent variable (Model 1) to multidimensional with two latent factors (Model 2) and three latent factors (Model 3), from a fully symmetrical bifactor solution (Model 4) with a General factor (G) and the three Specific factors (S) to a bifactor (S-1) solution (Model 5) with the contentment dimension considered as the General Reference Factor (GFR) and the inner-peace and fluctuating dimensions as Specific Residual Factors (SRF). Findings indicated better and adequate fit indices for the bifactor (S-1) solution compared to the other measurement models. Study 2 involving four samples was carried out to replicate and confirm the measurement model and to assess reliability, convergent and discriminant validity and temporal stability. Findings from Sample 1 confirmed the bifactor (S-1) structure, thus shedding more light on the internal construct of happiness. Indeed, omega values supported the internal reliability of the construct: the contentment factor could be considered as a broad general latent trait; similarly, the fluctuating and inner-peace factors exhibited strong specificity for their interested factors. This suggests that the durable contentment dimension may be considered as the GRF, since it refers to an optimal way of being and, therefore, to a *lasting* state of being that could be maintained through the upheavals of life [14]. The fluctuating and inner-peace dimensions can be interpreted as the SRF, as they contained a substantial amount of true score variance, above-and-beyond the general reference factor. In addition, the values of the PRV, which were obtained in the multidimensional composite at the factor level, together with the values of EVC and PUC at the model level provided further empirical evidence of the multidimensional conceptualization of the construct.

Notably, compared to the French version, the inner-peace and contentment subscales of the Italian version contained a reduced number of items equal to four and an increased number equal to 9, respectively. When observing the findings obtained from indices at the item level, a different correspondence between the inner-peace and contentment factors emerged: some items did not reflect the expected indicator, thus collapsing in a different factor. For instance, item 5 referred to peace of mind collapsed in the contentment factor, thus implying that its meaning is closer to the sense of durable happiness and satisfaction rather than to a feeling of being in harmony with oneself and others. Furthermore, item 9 related to beatitude collapsed in the inner-peace dimension, being more related to the sense of fullness and harmonious interconnectedness rather to pleasure and general well-being. Future research should replicate these results with other samples in order to confirm the internal structure of the two subscales. In spite of some internal changes between the two subscales, findings from sample 1 generally provided empirical evidence of the multidimensionality of the construct of happiness, a clear distinction of the two main dimensions of happiness and good levels of internal reliability and consistency for each scale and subscale. Finally, high indices of construct validity indicating how well latent variables were defined by underlying items, and high estimates of factor determinacy proved satisfying reliability of factor score measurement.

Consistent with French findings [14], results from samples 2 and 3 demonstrated adequate convergent validity of both scales. As expected, with regard to the associations between SA-DHS and well-being measures, bivariate and partial correlations supported the idea that this *lasting* state of happiness was more closely related to emotional positivity and eudaimonic well-being measures. On the other hand, the observed negative associations of the SFHS with positive aspects of well-being measures, as well as its positive links with negative affectivity and emotions indicated that such a swinging and unstable facet of happiness was to a lesser extent related to general life satisfaction and emotional positivity and more closely related to the hedonic principle of pleasure, which is by nature transitory.

In addition, findings from associations of the two aspects of happiness with optimism, mindfulness, the presence of and the search for meaning in life corroborated prior findings emerging with well-being measures. Indeed, as hypothesized and consistent with previous investigations [18,19,20], patterns of associations in opposite direction were found: the positive links of the authentic component with psychological constructs may lead to the assumption that such a facet of happiness could be considered as a further indicator of eudaimonic well-being and, therefore, definable as *eudaimonic* happiness. Likewise and in a speculative manner, the negative links of the fluctuating component with these constructs may reasonably suggest the need to consider such an aspect as an indicator of hedonic well-being, and definable as *hedonic* happiness, which is characterized by the wavering alternation of pleasure and displeasure phases.

The distinction between the two components of SA-DHS was also detected by the convergent analysis. Findings showed different patterns of association: while contentment proved to be theoretically related with well-being measures, inner-peace was positively related only to positive emotions and to the cognitive component of meaning in life. The inner peace-presence of meaning in life relationship, which emerged only in the current study and not in the French investigation, emphasized the crucial role played by inner-peace together with the presence of meaning in life, already identified as a well-being indicator [21,22,24], in healthy psychological functioning and in the perceived durable state of optimal being. In other words, individuals who comprehend and perceive their lives as significant and purposeful, experience feelings of being in harmony with themselves and others.

As regards the discriminant validity of the instrument, the findings partially supported previous research [25,45] which reported no association with age. In the current study, age was unrelated to authentic–durable happiness but weakly and negatively related to fluctuating happiness: younger adults seemed to show more variation in the alternation of pleasure and displeasure phases in line with LeFebvre and Huta [46] research. A possible explanation may be related to the data collection period. Indeed, significant even if weak associations were found in the samples recruited during the pandemic period, social distancing may well have caused meaningful life experiences and unstable emotions. In addition, it should also be borne in mind that the fluctuating dimension of happiness is subjected by nature to wavering moods. The significant differences in the mean scores of the same dimension found in the temporal stability analysis confirmed the swinging state of fluctuating happiness. In contrast, evidence of a more durable nature of authentic happiness was supported by the lack of significant differences in the mean scores of the dimension together with the correlation coefficient in line with the recommended value. Further studies are needed to better clarify the conflicting results on this topic.

The current research suffered from certain limitations. As an overall assessment, findings showed an adequate fitting measurement model, the multidimensionality of the instruments and good levels of internal reliability. However, further research should explore measurement invariance across age and gender. Although the two dimensions of happiness were clearly distinct, the two subscales of the authentic–durable happiness displayed a different internal consistency in comparison with the French version. Consequently, further conceptual improvements of the subscales, for example by using constructs closer to inner-peace such as social connectedness, could develop more refined measures. Future studies should attempt to replicate current findings with more representative Italian samples, recruited through random sampling and more diversified in terms of demographic features that may be differently related to the way of experiencing fluctuating and durable happiness. Another limitation of the current research concerned the lack of empirical evidence of the SSHM theoretical model that should also be examined.

Finally, longitudinal assessment including the experience sampling method or experimental design could be applied to infer causal and temporal relationships between experienced happiness and psychological constructs.

Some theoretical and practical implications should be noted. First, further research is needed to assess the Self-centeredness/Selflessness Happiness Model (SSHM) underlying the conceptualization of the two facets of happiness. Second, in light of the increasing academic interest, it is important to consider the implications of the bifactor modelling. Prior studies generally explored the construct of happiness based on a unidimensional level/perspective without taking into account simultaneously both the inter- and intraindividual variability. However, despite the recognized significance of testing multifaceted constructs of happiness with an appropriate approach, limited research has been addressed to this issue. Applying a bifactor modelling approach, the current study sought to assess the unique and distinct contributions of the facets of happiness (from fluctuating and to inner-piece dimension) as well as the general construct (e.g., contentment) in order to separate these contributions from the effects of the general construct. This approach could shed more light on inter- and intraindividual differences in experienced happiness. Collectively, findings indicated how the two distinct dimensions tended to relate to external variables in a different way also showing demographic variations. When bearing in mind these individual differences, an incremental contribution of the specific factors, over and beyond the general factor, should be tested in relation to external health variables such as Psychological General Well-Being Indices [47], or to poor health outcomes.

This study may represent a first step towards broadening the focus of happiness research to build a shared conceptual background and understand optimal human functioning beyond individual specificities. To be healthy and have a good quality of life involves being happy in an authentic way!

## Figures and Tables

**Table 1:** Means and standard deviations for all the scores of the scales in sex and age groups.

	SFHS	SA–DHS	Contentment	Inner-Peace
	Mean (SD)	Mean (SD)	Mean (SD)	Mean (SD)
Males (*n* = 261)	3.89 (1.11)	4.48(1.02)	4.91 (1.02)	4.72 (1.20)
Females (*n* = 283)	4.41 (1.32)	4.49 (1.07)	4.59 (1.03)	4.30 (1.30)
Adolescents (*n* = 258)	4.12 (1.19)	4.74 (1.03)	4.86 (1.01)	4.56 (1.27)
Adults (*n* = 268)	4.19 (1.30)	4.56 (1.08)	4.64 (1.05)	4.49 (1.26)
Males (*n* = 261)				
Adolescents (*n* = 156)	3.83 (1.06)	4.97 (1.00)	5.05 (0.98)	4.85 (1.22)
Adults (*n* = 105)	3.99 (1.17)	4.63 (1.04)	4.71 (1.04)	4.54 (1.14)
Females (*n* = 283)				
Adolescents (*n* = 102)	4.57 (1.25)	4.39 (0.99)	4.57 (0.97)	4.10 (1.21)
Adults (*n* = 181)	4.30 (1.36)	4.55 (1.10)	4.60 (1.06)	4.46 (1.33)

**Table 2:** Fit Indexes of the measurement models.

	YBχ^2^	*df*	*p*	RMSEA (90% C.I.)	CFI	SRMR
Model 1	2092.61	230	<0.001	0.122 (0.117–0.127)	0.612	0.136
Model 2	878.380	229	<0.001	0.072 (0.067–0.077)	0.865	0.058
Model 3	774.121	227	<0.001	0.067 (0.061–0.072)	0.886	0.056
Model 4	533.788	207	<0.001	0.054 (0.048–0.060)	0.932	0.050
Model 5	663.422	214	<0.001	0.062 (0.057–0.068)	0.906	0.046

Model 1 = Unidimensional solution; Model 2 = Two oblique factors; Model 3 = Three oblique. factor; Model 4 = Fully Symmetrical Bifactor; Model 5 = Bifactor (S-1).

**Table 3:** Standardized loadings factor of bifactor confirmatory factor analysis for the general latent trait (GRF) and its specific residual latent traits.

	GRF	SRF1	SRF2	I-EVC
Fluctuating dimension				
Item 1	−0.135	0.202		0.309
Item 2	−0.274	0.383		0.339
Item 3	−0.300	0.539		0.237
Item 4	−0.228	0.565		0.140
Item 5	−0.275	0.716		0.129
Item 6	−0.285	0.572		0.199
Item 7	−0.379	0.698		0.228
Item 8	−0.307	0.698		0.162
Item 9	0.209	0.557		0.123
Item 10	0.158	0.543		0.078
Authentic–durable dimension				
Item 1	0.687			1.000
Item 2	0.778			1.000
Item 3	0.726			1.000
Item 4	0.729			1.000
Item 5	0.59		0.623	1.000
Item 6	0.687			0.473
Item 7	0.689		0.400	1.000
Item 9	0.749			0.748
Item 10	0.562		0.618	1.000
Item 11	0.675			0.453
Item 12	0.793			1.000
Item 14	0.503		0.601	1.000
Item 15	0.753		0.166	0.412

GRF = General Reference Factor, Contentment; SRF1 = Specific Residual Factor 1, Fluctuating; SRF2 = Specific Residual Factor 2, Inner-peace; I-ECV = Item Explained Common Variance; Item 8, 13 and 16 are not included in the table, because they measure durable unhappiness.

**Table 4:** Ancillary Bifactor Evaluation Indices.

Indices	GRF	SRF1	SRF2
EVC	0.605	0.282	0.113
EVC New	0.605	0.317	0.157
ω/ωS	0.880	0.857	0.895
Hω/HωS	0.520	0.308	0.055
Relative omegas	0.589	0.822	0.357
H index	0.932	0.844	0.668
FD	0.960	0.923	0.868
α	0.896	0.857	0.837

EVC = Explained Common Variance; EVC New = Explained Common Variance; ω = Omega; ωS = Omega subscale; Hω = Omega Hierarchical; HωS = Omega Hierarchical Subscale; FD = Factor determinacy; α = Internal consistency.

**Table 5:** Zero-order and partial correlations of the Subjective Authentic–Durable Happiness dimension, the Subjective Fluctuating Happiness dimension, Contentment and Inner peace dimension with various measures in Sample 2.

	LOT-R	NEGA EMO	POS EMO	POM	SFM	MAAS
Bivariate correlations in the total sample (*N* = 254)						
SFHS	−0.485 ** (−0.378 **)	0.392 ** (0.305 **)	−0.248 ** (−0.071)	−0.292 ** (−0.136 *)	0.292 ** (0.236 **)	−0.231 ** (−0.151*)
SA-DHS	0.425 ** (0.286 **)	−0.316 ** (−0.187 **)	0.472 ** (0.419 **)	0.447 ** (0.376 **)	−0.199 ** (−0.092)	0.240 ** (0.165 **)
Bivariate correlations in males (*n* = 115)						
SFHS	−0.486 ** (−0.398 **)	0.387 ** (0.323 **)	−0.139 (0.018 **)	−0.310 ** (−0.177)	0.266 ** (0.218*)	−0.151 (−0.093)
SA-DHS	0.434 ** (0.325 **)	−0.278 ** (−0.168)	0.449 ** (0.432 **)	0.476 ** (0.414 **)	−0.189 ** (−0.108)	0.189 * (0.148)
Bivariate correlations in females (*n* = 139)						
SFHS	−0.347 ** (−0.252 **)	0.273 ** (0.193 *)	−0.209 ** (−0.058)	−0.253 ** (−0.121)	0.136 ** (0.103 **)	−0.342 ** (−0.266 **)
SA-DHS	0.349 * (0.255 **)	−0.276 ** (−0.197 *)	0.443 ** (0.403 **)	0.411 ** (0.355 **)	−0.114 (−0.070)	0.288 ** (0.187 *)
Partial correlations in the total sample						
1 Factor C	0.395 ** (0.114 **)	−0.343 ** (−0.284 **)	0.428 **(0.079)	0.395 **(0.027)	−0.180 ** (−0.031)	0.242 ** (0.136 *)
2 Factor I	0.421 ** (0.194 **)	−0.219 ** (0.088)	0.489 ** (0.273 **)	0.483 ** (0.304 **)	−0.205 ** (−0.105)	0.203 ** (0.021)
Partial correlations in males						
1 Factor C	0.426 ** (0.243 **)	−0.348 ** (−0.409 **)	0.411 ** (0.145)	0.428 ** (0.126)	−0.184 * (−0.094)	0.185 * (0.098)
2 Factor I	0.372 ** (0.097)	−0.097 (−0.298 **)	0.440 ** (0.223*)	0.481 ** (0.273 **)	−0.165 (−0.045)	0.163 (0.041)
Partial correlations in females						
1 Factor C	0.309 ** (−0.008)	−0.280 ** (−0.158)	0.395 ** (0.007)	0.354 ** (−0.058)	−0.089 (0.054)	0.293 ** (0.170)
2 Factor I	0.385 *** (0.242 **)	−0.234 ** (−0.011)	0.481 ** (0.299 **)	0.471 ** (0.337 **)	−0.147 (−0.129)	0.242 ** (0.007)

*** *p* < 0.001; ** *p* < 0.010; * *p* < 0.050; 1 factor C = Contentment; 2 factor I = Inner-peace; SFHS = Subjective Fluctuating happiness Scale; SA-DHS = Subjective Authentic durable Happiness Scale; LOT-R = Dispositional Optimism; NEGA EMO = Negative Emotions, POS EMO = Positive Emotion; POM = Presence of Meaning; SFM = Search for Meaning; MAAS = Mindful Attention Awareness Scale.

**Table 6:** Zero-order and partial correlations of the Subjective Authentic–Durable Happiness Scale and the Subjective Fluctuating Happiness Scale with well-being and happiness measures in Sample 3.

	LS	OHI	FS	SPANE POS	SPANE NEG
Bivariate and partial correlations in the total sample (*N* = 488)					
SFHS	−0.255 ** (−0.253 **)	−0.245 ** (−0.246 **)	0.133 ** (−0.119 *)	−0.233 ** (−0.225 **)	0.400 ** (−0.394 **)
SA−DHS	0.037 (0.015)	0.000 (−0.022)	0.194 ** (0.184 **)	0.112 * (0.094)	−0.140 ** (−0.115 *)
Bivariate and partial correlations in adolescents (*n* = 367)					
SFHS	−0.294 ** (−0.302 **)	−0.266 ** (−0.267 **)	−0.139 ** (−0.156 *)	−0.239 ** (−0.248 **)	0.431 ** (0.437 **)
SA−DHS	0.050 (−0.090)	−0.012 (0.021)	0.128 * (0.147 **)	0.054 (0.086)	−0.024 (−0.085)
Bivariate and partial correlations in young adults (*n* = 121)					
SFHS	−0.190 * (−0.127)	−0.235 ** (−0.181 *)	−0.067 (−0.006)	−0.229 * (−0.172)	0.291 ** (0.252 **)
SA−DHS	0.552 ** (0.539 **)	0.587 ** (0.574 **)	0.399 ** (0.395 **)	0.698 ** (0.689 **)	−0.451 ** (−0.430 **)
Bivariate and partial correlations in males (*n* = 220)					
SFHS	−0.321 ** (−0.325 **)	−0.311 ** (−0.310 **)	−0.262 ** (−0.258 **)	−0.288 ** (−0.287 **)	0.379 ** (0.376 **)
SA−DHS	−0.048 (−0.070)	0.033 (0.016)	0.121 (0.110)	0.027 (0.011)	−0.090 (−0.074)
Bivariate and partial correlations in females (*n* = 268)					
SFHS	−0.183 ** (−0.156 *)	−0.157 ** (−0.141 *)	−0.053 (−0.006)	−0.176 ** (−0.134 *)	0.403 ** (0.368 **)
SA−DHS	0.149 * (0.115)	0.090 (0.059)	0.218 ** (0.212 **)	0.227 ** (0.197 **)	−0.256 ** (−0.190 **)
Bivariate and partial correlations in age*gender					
Bivariate and partial correlations in adolescent males (*n* = 203)					
SFHS	−0.362 ** (−0.199 **)	−0.317 ** (−0.151 **)	−0.269 ** (−0.094)	−0.304 ** (−0.143 *)	0.373 ** (0.318 **)
SA−DHS	0.705 ** (0.667 **)	0.640 ** (0.598 **)	0.611 ** (0.575 **)	0.606 ** (0.564 **)	−0.255 ** (−0.153 *)
Bivariate and partial correlations in adolescent females (*n* = 164)					
SFHS	−0.156 * (−0.166 **)	−0.129 (−0.128)	−0.001 (−0.016)	−0.108 (−0.119)	0.463 ** (0.478)
SA−DHS	0.075 (0.095)	−0.012 (0.003)	0.136 (0.137)	0.092 (0.105)	−0.084 (−0.155 *)
Bivariate and partial correlations in young adult males (*n* = 17)					
SFHS	0.149 (0.075)	−0.206 (−0.332)	−0.075 (−0.109)	0.016 (−0.182)	0.463 (0.534 **)
SA−DHS	0.400 (0.362)	0.428 (0.491)	0.150 (0.170)	0.703 ** (0.715 **)	−0.225 (−0.369)
Bivariate and partial correlations in young adult females (*n* = 104)					
SFHS	−0.237 * (−0.164)	0.235 * (−0.159)	−0.067 (0.012)	−0.246 * (−0.169)	0.273 ** (0.215)
SA−DHS	0.579 ** (0.382)	0.603 ** (0.492)	0.424 ** (0.170)	0.698 ** (0.715 **)	−0.478 ** (−0.369)

** *p* < 0.010; * *p* < 0.050; SFHS = Subjective Fluctuating happiness Scale; SA-DHS = Subjective Authentic durable Happiness Scale; LS = Life satisfaction; OHI = Oxford Happiness Inventory; FS = Flourishing; SPANE POS = Positive Affectivity; SPANE NEGA = Negativity Affectivity.

**Table 7:** Zero-order and partial correlations of the two factors, Contentment and Inner-peace, with well-being and happiness measures in Sample 3.

	LS	OHI	FS	SPANE POS	SPANE NEG
Bivariate and partial correlations in the total sample (N = 488)					
1 Factor C	0.613 ** (0.264 **)	0.615 ** (0.287 **)	0.513 ** (0.249 **)	0.656 ** (0.391 **)	−0.338 ** (−0.231 **)
2 Factor I	0.628 ** (0.316 **)	0.612 ** (0.283 **)	0.482 ** (0.174 **)	0.591 ** (0.190 **)	−0.257 ** (0.007)
Bivariate and partial correlations in adolescents (*n* =367)					
1 Factor C	0.657 ** (0.329 **)	0.638 ** (0.294 **)	0.578 ** (0.328 **)	0.649 ** (0.372 **)	−0.306 ** (−0.221 **)
2 Factor I	0.637 ** (0.272 **)	0.634 ** (0.291 **)	0.508 ** (0.129 *)	0.588 ** (0.182 **)	−0.224 ** (0.031)
Bivariate and partial correlations in young adults (*n* =121)					
1 Factor C	0.493 ** (0.098)	0.555 ** (0.267 **)	0.357 ** (0.072)	0.680 ** (0.443 **)	−0.448 ** (−0.228 **)
2 Factor I	0.604 ** (0.411 **)	0.553 ** (0.262 **)	0.430 ** (0.265 **)	0.600 ** (0.210 **)	−0.362 ** (−0.060)
Bivariate and partial correlations in males (*n* =220)					
1 Factor C	0.664 ** (0.368 **)	0.599 ** (0.279 **)	0.588 ** (0.363 **)	0.596 ** (0.324 **)	−0.260 ** (−0.214 **)
2 Factor I	0.622 ** (0.246 **)	0.589 ** (0.263 **)	0.488 ** (0.104)	0.547 ** (0.185 **)	−0.176 ** (0.059)
Bivariate and partial correlations in females (*n* =268)					
1 Factor C	0.575 ** (0.196 *)	0.625 ** (0.298 **)	0.493 ** (0.200 **)	0.687 ** (0.431*)	−0.377 ** (−0.235 **)
2 Factor I	0.628 * (0.306 **)	0.624 ** (0.297 **)	0.502 ** (0.227 **)	0.614 ** (0.197 **)	−0.304 ** (−0.033)
Bivariate and partial correlations in age*gender					
Bivariate and partial correlations in adolescent males (*n* =203)					
1 Factor C	0.691 ** (0.412 **)	0.606 ** (0.272 **)	0.620 ** (0.385 **)	0.591 ** (0.314 **)	−0.264 ** (−0.209 **)
2 Factor I	0.629 ** (0.226 **)	0.606 ** (0.287 **)	0.519 ** (0.124 **)	0.546 ** (0.190 **)	−0.187 ** (−0.048)
Bivariate and partial correlations in adolescent females (*n* =164)					
1 Factor C	0.611 ** (0.239 **)	0.671 ** (0.333 **)	0.568 ** (0.298 **)	0.694 ** (0.430 **)	−0.315 ** (−0.223 **)
2 Factor I	0.635 ** (0.321 **)	0.659 ** (0.294 **)	0.525 ** (0.160*)	0.618 ** (0.172 **)	−0.228 ** (0.026)
Bivariate and partial correlations in young adults males (*n* = 17)					
1 Factor C	0.302 (−0.19)	0.454 (0.323)	0.122 (−0.002)	0.708 ** (0.499 **)	−0.271 (−0.271)
2 Factor I	0.496 * (0.427)	0.337 (−0.004)	0.165 (0.112)	0.589* (0.128)	−0.124 (0.123)
Bivariate and partial correlations in young adults females (*n* = 104)					
1 Factor C	0.525* (0.135)	0.564 ** (0.248 **)	0.380 ** (0.071)	0.679 ** (0.436)	−0.467 ** (−0.281)
2 Factor I	0.621 ** (0.409 **)	0.584 ** (0.307 **)	0.461 ** (0.292 **)	0.605 ** (0.220)	−0.399 ** (−0.096)

** *p* < 0.010; * *p* < 0.050; 1 Factor C = Contentment; 2 Factor I = Inner-peace; LS = Life satisfaction; OHI = Oxford Happiness Inventory; FS = Flourishing; SPANE POS = Positive Affectivity; SPANE NEGA = Negativity Affectivity.

## Data Availability

The data presented in this study are available on request from the corresponding author

## Appendix A

**Table A1:** The Italian version of the happiness scales.

*Fluctuating dimension* (SFHS) Wthin the past few months./*Negli ultimi mesi*,
1 I have had satisfactions and also great disappointments *Ho avuto soddisfazioni, ma anche grandi insoddisfazioni*
2 The periods of pleasure that I have known are always followed by periods of displeasure *I periodi di piacere che ho conosciuto sono stati sempre seguiti da periodi di dispiacere*
3 My level of serenity is very changeable *Il mio livello di serenità è molto variabile.*
4 I have often known periods of euphoria but they are almost always followed by much less exciting periods *Ho conosciuto spesso periodi di euforia, che sono stati seguiti, però, quasi sempre da periodi molto meno esaltanti*.
5 I often go from euphoria to sadness. *Spesso passo dall’euforia alla tristezza*
6 Periods of ill-being follow periods of well-being *A periodi di benessere seguono periodi di malessere.*
7 My level of happiness is rather unstable, sometimes high, sometimes low *Il mio livello di felicità è piuttosto instabile, a volte alto, a volte basso*.
8 I often go from a rather high level of pleasure to a rather low level of pleasure *Spesso passo da un livello di piacere piuttosto alto ad un livello piuttosto basso*.
9 I have times when I swing from moments of total bliss to much less satisfying moments *Conosco delle alternanze tra momenti di beatitudine totale a momenti molto meno soddisfacenti*.
10 In the same day, I can sometimes be happy and sometimes sad *Nello stesso giorno mi può succedere di essere a volte felice e a volte infelice*.
*Authentic–durable dimension* (SA-DHS) In your life, what is your regular level of/*Nella tua vita, indica il tuo livello generale di*
1 Overall well-being? *Benessere generale*
2 Happiness? *Felicità*
3 Pleasure? *Piacere*
4 Bliss? *Felicità che sembra completa*
5 Peace of mind? *Tranquillità d’animo*
6 Satisfaction? *Soddisfazione*
7 Serenity? *Serenità*
9 Beatitude? (perfect happiness)? *Beatitudine, Felicità perfetta*
10 Inner-peace? *Pace interior*
11 Fulfillment? *Appagamento*
12 Joy? *Gioia*
14 Tranquility (inner-calm)? *Calma interiore*
15 Plenitude (feeling of complete satisfaction, happiness and fulfillment)? *Pienezza, ossia sentimento di completa soddisfazione e realizzazione*

Note: Item 8 Displeasure (*Dispiacere*), 13 Feeling bad (*Malessere*) and 16 Unhappiness (*Infelicità*) were deleted from SA-DHS, since they assess durable unhappiness.
